# Neural Networks and Fault Probability Evaluation for Diagnosis Issues

**DOI:** 10.1155/2014/370486

**Published:** 2014-07-15

**Authors:** Yahia Kourd, Dimitri Lefebvre, Noureddine Guersi

**Affiliations:** ^1^Department of Control Engineering, University of Mohamed Khider, 07000 Biskra, Algeria; ^2^Electrical Engineering and Automatic Control Research Group (GREAH), University of Le Havre, 25 rue Philippe Lebon, 76058 Le Havre, France; ^3^Department of Electronics, University of Badji Mokhtar, 23000 Annaba, Algeria

## Abstract

This paper presents a new FDI technique for fault detection and isolation in unknown nonlinear systems. The objective of the research is to construct and analyze residuals by means of artificial intelligence and probabilistic methods. Artificial neural networks are first used for modeling issues. Neural networks models are designed for learning the fault-free and the faulty behaviors of the considered systems. Once the residuals generated, an evaluation using probabilistic criteria is applied to them to determine what is the most likely fault among a set of candidate faults. The study also includes a comparison between the contributions of these tools and their limitations, particularly through the establishment of quantitative indicators to assess their performance. According to the computation of a confidence factor, the proposed method is suitable to evaluate the reliability of the FDI decision. The approach is applied to detect and isolate 19 fault candidates in the DAMADICS benchmark. The results obtained with the proposed scheme are compared with the results obtained according to a usual thresholding method.

## 1. Introduction

Industrial complex automated systems are vulnerable to many types of faults (due to sensors, actuators, components, etc.). In order to maintain normal operating conditions, the human operator plays the roles of supervisor according to the several plant parameters, measurements, and observations. These faults may be abrupt or incipient. Due to the growing complexity of modern engineering systems and ever increasing demand for safety and reliability, there is a great interest in the development of fault detection and isolation (FDI) methods. Those techniques are important in process engineering because plant faults may cause abnormal operations and, if not detected early, can cause emergency shutdowns and also definitive damages. Moreover, the quality of production will not be maintained in abnormal situations (i.e., process variables deviate significantly from their nominal values). Therefore, designing robust FDI systems has received considerable attention both from industry and academia [1]. The robustness of the method depends mainly on the reliable discrimination between the effects of uncertainties in the model behavior, noises in the signal measurements, and faults that may occur [2].

FDI methods are generally separated into model-based and data-based approaches. The advantage of model-based approaches is to lead easily to residual signals by comparing the behaviors of the system with the model and to provide a mathematical framework that can be used to evaluate the performance of the method [3–5]. For nonlinear systems, the standard approach is to linearize the model around the operating point and to make use of usual contributions derived from linear system theory. However, linearization does not always provide a good model for the processes, in particular when strongly nonlinear behaviors are observed. Moreover, complex processes often operate in multiple operating regimes in industrial applications (e.g., mining, chemical treatment, and water treatment). So it is often not possible to obtain linear models that accurately describe the plants in all regimes. One solution is to use nonlinear methods such that nonlinear observers with analytical approach and geometric approach which require a perfect knowledge of nonlinear system [6–8]. But, nonlinear observers are limited to a few types of standard nonlinearities. Furthermore, the nonlinear observer approach can be used only when the nonlinear systems dynamics are known with sufficient confidence; this is rarely the case for real system applications [2, 4, 9, 10]. To solve the nonlinear problem of observed data, nonlinear PCA (principal component analysis) and PLS (partial least squares) approaches have been developed [11, 12]. However, PCA and PLS have a linearity assumption, limiting their application.

An attractive alternative to nonlinear techniques is to use linear multimodel strategies. The multimodel approach has been often used in recent years for the modeling and control of nonlinear systems [13]. Multimodel methods for FDI are based on the partition of the operating range into separate regions [14]. Local linear models are applied in each region. It has also been associated with Kalman filters in order to detect, isolate, and estimate the state of a system in presence of faults [3, 15, 16]. In addition, Lane et al. proposed a multigroup model to monitor batch processes with multiple modes [17]. Hwang and Han assumed that different operating modes have the same number of retained principal components and proposed a super PCA model to monitor multimode batch processes [18]. More recently, effectiveness of the multimodel approaches for FDI of real industrial systems has been discussed [14, 19–21] and Baniardalani et al. proposed a qualitative model based on fault diagnosis using a threshold level [22].

The main motivation for this research is to explore the potential of computational intelligence (CI) approaches to design models of faulty behaviors and to generate residuals for nonlinear systems [23–26]. Diagnosis is a complex reasoning activity, which is currently one of the domains where artificial intelligence techniques have been successfully applied as these techniques use association rules, reasoning, and decision making processes as would the human brain in solving diagnostic problems. The proposed method combines the benefits of model-based method (to easily generate residuals) with those of data-based methods (probabilistic methods for isolation). Some methods have been developed based on neural networks (NNs) [27]. Kramer developed a nonlinear PCA based on autoassociative neural networks having five layers [28]. Chen and Liao proposed dynamic process fault monitoring based on neural network and PCA [29]. The NN approaches are regarded as multivariate nonlinear analytical tools capable of recognizing patterns from noisy complex data. Their major advantages include learning, noise suppression, and parallel data processing [10].

Intelligent systems found broad application in fault diagnosis from their early stages because an expert system simulates human reasoning about a problem domain, performs reasoning over representations of human knowledge, and solves problems using heuristic knowledge rather than precisely formulated relationships, in forms that reflect more accurately the nature of most human knowledge. Neural networks are able to learn diagnostic knowledge from process operation data. However, the learned knowledge is in the form of weights which are difficult to comprehend.

In this work, an FDI method is proposed that generates a large number of residuals computed according to the set of fault candidates. For each fault candidate, a model of faulty behavior is worked out and residuals are obtained with this model. The advantage of using models for both fault-free and faulty behaviors lies in the fact that, in addition to estimating the state of the system, faulty models provide the probability of occurrence or activation of each model in case of dysfunction. These probabilities are used for diagnosis issues. The residuals are analyzed according to their magnitude and signature and a confidence factor evaluates the performance of the decision. The method is validated with the DAMADICS benchmark process [30]. This benchmark is well-defined for FDI purposes. The paper is organized as follows: In Section 2, the FDI problem is presented for the DAMADICS valve actuator. In Section 3, the design of NN models for faulty and fault-free behaviors is put forward and FDI based on those models is developed in Section 4.  Section 5 presents the application of our contributions to the DAMADICS benchmark problem. Finally, in Section 6, some concluding remarks are provided.

## 2. FDI for Electropneumatic Actuator

The DAMADICS benchmark is an engineering research case-study that can be used to evaluate FDI methods. The benchmark is an electropneumatic valve actuator in the Lublin sugar factory in Poland [30]. The DAMADICS has been used as test bed of the fault detection and diagnosis approach proposed in this paper. Its main characteristics are as follows:

- the DAMADICS benchmark is based on the physical phenomena that give origin to faults in the system;
- the DAMADICS benchmark clearly defines the process and data sets; the fault scenarios are standardized. This is done in view of industrial applicability of the tested FDI solutions, to cut off methods that have no practical feasibility.

### 2.1. Electropneumatic Actuator Description

**Figure 1:**
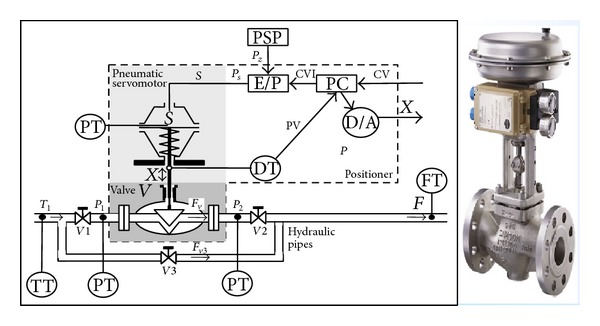
Structure of DAMADICS actuator system.

The actuator consists of three main parts as follows: control valve (*V*); pneumatic servomotor (*S*); positioned (*P*). It is depicted in Figure 1. Furthermore, each of the three main parts consists of other components shown in Figure 1, such as the following: positioner supply air pressure (PSP); PT: air pressure transmitter; FT: volume flow rate transmitter; TT: temperature transmitter; ZT: rod position transmitter; E/P: electropneumatic converter; V1, V2: cut-off valves; V3: by-pass valve; Ps: pneumatic servomotor chamber pressure; and CVI: controller output (PC output). In this actuator, faults can appear in control valve, servomotor, electropneumatic transducer, piston rod travel transducer, pressure transmitter, or microprocessor control unit. A total number of 19 types different faults are considered (*p* = 19, Table 1). The faults are emulated under carefully monitored conditions, keeping the process operation within acceptable limits. Five available measurements and one control value signal have been considered for benchmarking purposes: process control external signal (CV), liquid pressures on the valve inlet (*P*_1_) and outlet (*P*_2_), liquid flow rate (*F*), liquid temperature (*T*_1_), and servomotor rod displacement (*X*) (Table 2).

To test the robustness of the proposed fault detection and diagnosis method, several tests have been performed with the set of 19 different types of abrupt and incipient faults with several severities, according to the benchmark rules defined in the actuator benchmark library (DABLib) [31]. The simulations have been conducted considering the physical variables free of noise and affected by noise. Furthermore, all simulation tests have been performed considering the simulator input variables. A sampling time of 1 s has been used by the fault detection system, while the simulator uses a fourth-order Runge-Kutta method with a fixed step size of 0.0025 s. The results achieved during the tests are summarized in Table 1. The white cells in Table 1 indicate that such faulty scenarios were not considered for benchmark purposes.

Within the DAMADICS project the actuator simulator was developed under MATLAB Simulink. This tool makes it possible to generate data for the normal operating mode and also for the 19 faulty modes. The considered faults are presented in Table 1. They can be considered either as abrupt or incipient. Abrupt faults may have small (S), medium (M), or big (B) magnitude. The mark “∗” denotes the faults that are specified for benchmark. In this study, results are provided in case of big magnitude.

### 2.2. FDI Issues for Electropneumatic Actuator

The conditions for testing and validating the FDI algorithms on the actuator benchmark are given in [32, 33]. The system has already experimented several FDI methods [34–36]. In [36], binary-valued evaluation of the fault symptoms is explored and the authors focus on the optimization of the neural network architecture according to Akaide Information Criteria and Final Prediction Error. Both criteria include the learning error and also a term that depends on the complexity (size of the network in number of nodes) and on the dimension of the learning set in order to optimize the ratio complexity/performance. The authors provide interesting performances with small networks for detection but some faults are not isolable. In comparison, our approach will require a larger number of networks and the networks have more nodes but all faults will be detected and isolated. In [34], multiple-valued evaluation of the fault symptoms is introduced to improve the isolation of faults. Such a method requires a heuristic knowledge about influence of faults on residuals. In comparison, our approach uses 3-valued evaluation of the residuals for fault-free behaviors and binary-valued evaluation of the residuals for faulty behaviors.

## 3. Design of Models for Faulty and Fault-Free Behaviors

### 3.1. Model of Fault-Free Behaviors

Physical processes are very often complex dynamic systems, having strong nonlinearities. As a consequence, knowledge based models are not easy to obtain. Simplifications are essential to formulate an exploitable model, but are degrading the accuracy of the mathematical model. Other problems remain with some model parameters that are not easy to measure or estimate and that could be variable in time. Another approach lies in the systematic processing of data collected by sensors.

At this stage, unknown nonlinear systems are considered with input vector *U*(*t*) = (*u*_*i*_(*t*)), *i* = 1,…, *q*, and output vector *Y*(*t*) = (*y*_*k*_(*t*)), *k* = 1,…, *n*. The state variables are not measurable. NNs are introduced to generate accurate models of the system in normal operating conditions [37, 38]. The comparison between the output of the system and the output *Y*_0_′(*t*) = (*y*_*k*0_′(*t*)), *k* = 1,…, *n*, of the NN model gives the error vector *E*(*t*) = (*e*_*k*_(*t*)), *k* = 1,…, *n*, with

(1) $$\begin{array}{c} e_{k}\left(t\right)=y_{k}\left(t\right)-y_{k0}^{\prime }\left(t\right). \end{array}$$

The learning of the ANN is obtained according to the Levenberg-Marquardt algorithm with early stopping. This algorithm is known for its rapid convergence. During learning stage, the NN is trained with data collected during the normal functioning of the system. The NN is then validated with another set of data. In order to get the best model, several configurations are tested according to a trial error processing that uses pruning methods to eliminate the useless nodes. Finally the resulting NN will be used as a fault-free model of the system.

### 3.2. Model of Fault-Free Behaviors for Actuator

We have constructed a multilayer perceptron (MLP) NN to model the coupled outputs *y*_1_(*t*) = *X*(*t*) and *y*_2_(*t*) = *F*(*t*) of the DAMADICS actuator system in case of fault-free behaviours. We note *y*_10_′(*t*) = *X*′(*t*) and *y*_20_′(*t*) = *F*′(*t*) the estimated values of *X*(*t*) and *F*(*t*) processed by the NNs:

(2) $$\begin{array}{c} \left(X^{\prime },F^{\prime }\right)=\text{NNFM}\left(0\right), \end{array}$$

where NNFM(0) stands for the double MLP structures with inputs CV, *P*_1_, *P*_2_, *T*_1_, *X*, *F*. To select the structure of NNFM(0), several tests have been carried out to obtain the best architectures (with minimal number of hidden layers and number of neurons by layer) for modeling the operation of the actuator.  Table 3 provides some results obtained during this stage. The training and test data were generated by the simulation of the Matlab Simulink actuator model. Validation is done by the measured data provided by the “Lublin Sugar Factory.”

From Table 3, the structure NNFM(0) = NNFM(6, 3, 2) is selected to avoid the phenomenon of overlearning. Adding more nodes in hidden layers does not improve the performance of NNFM(0).

**Figure 2:**
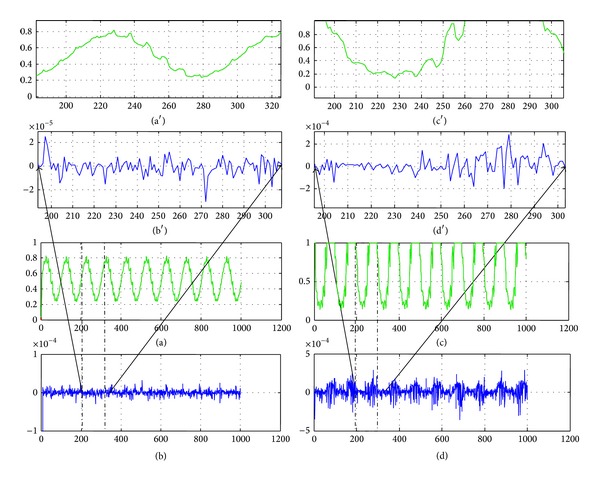
NNFM(0) validation (*X*-label: time (s), *Y*-label: output magnitude): (a) and (a′) actual output *y*_1_ and estimated output *y*_10_′; (b) and (b′) modeling error *e*_1_; (c) and (c′) actual output *y*_2_ and estimated output *y*_20_′; (d) and (d′) modeling error *e*_2_.

The system outputs *X* and *F* and estimated outputs *X*′ and *F*′ are reported in the Figures 2(a) and 2(c). The modeling errors *X*-*X*′ and *F*-*F*′ are reported in Figures 2(b) and 2(d). The modeling results are very satisfactory because no noise was considered and the modeling errors are less than 10^−5^ for the first output and about 10^−4^ for the second output.

### 3.3. Models of Faulty Behaviors

**Figure 3:**
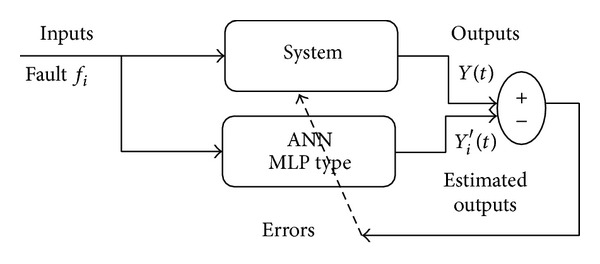
Design of NN models for faulty behaviors.

When multiple faults are considered, the isolation of the detected faults is no longer trivial and early diagnosis becomes a difficult task. One can multiply the measurements and use some analysis tools (residuals analysis) in order to isolate the faults. But the number of sensors limits the use of such approach. Another approach is to use a history of collected data to improve the knowledge about the faulty behaviors and then to use this knowledge to design models of faulty behaviors and additional residuals. Such models will be used to provide estimations for each fault candidate and then the decision results from the comparison of the estimations with the measurements collected during system operations. The systematic design of models for the fault-free behaviors is the first component of the proposed approach. The design of models for faulty behaviors is similar to the method described in Section 3.1. The learning of faulty behaviors is obtained according to the Levenberg-Marquardt algorithm with early stopping. Each model is built for a specific fault candidate *f*_*i*_ that is considered as an additional input.  Figure 3 exhibits the general scheme used to design a model of faulty behaviors. The vectors *Y*_*i*_′(*t*) = (*y*_*ki*_′(*t*)), *k* = 1,…, *n*, *i* = 1,…, *p* stand for the outputs of the NN models designed for the faults *f*_*i*_, *i* = 1,…, *p*.

### 3.4. Models of Faulty Behaviors for DAMADICS

**Figure 4:**
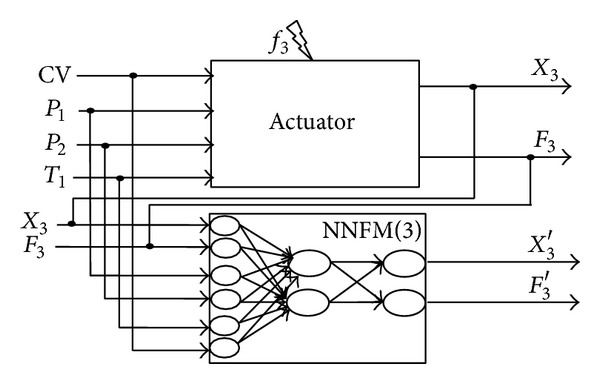
NN model NNFM(3) for fault *f*_3_.

The preceding method is applied to build NNs models corresponding to the 19 fault candidates that are considered with DAMADICS benchmark. For that purpose, it is necessary to create a data base that contains samples for all faults exposed to the DAMADICS system [39]. The method is illustrated in Figure 4 for the fault *f*_3_. The network NNFM(3) learns the mapping from *q* = 6 inputs to *n* = 2 outputs when fault *f*_3_ is assumed to affect the system from time *t* = 0. Equation (3) holds:

(3) $$\begin{array}{c} \left(X_{3}^{\prime },F_{3}^{\prime }\right)=\text{NNFM}\left(3\right), \end{array}$$

where NNFM(3) stands for the double MLP structures with inputs CV, *P*_1_, *P*_2_, *T*_1_, *X*, *F*. To select the structure of NNFM(3), numerous tests have been carried out to obtain the best architectures. The training and test data were generated using Matlab-Simulink DABLIB models (DAMADICS 2002). The best structure is a NN with 6 nodes in the first hidden layer, 3 nodes in the second hidden layer, and two output neurons. Validation is done with the measured data provided by the Lublin Sugar Factory in 2001 (DAMADICS 2002).

## 4. FDI with Models for Faulty and Fault-Free Behaviors

### 4.1. Principle

**Figure 5:**
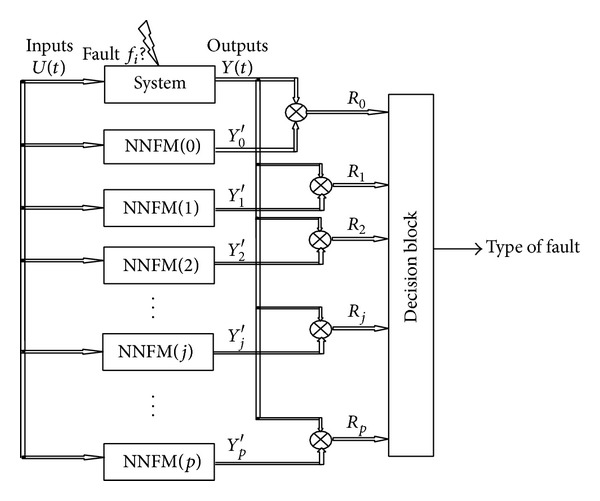
FDI design with models of faulty and fault-free behaviors.

The proposed approach is based on the analysis of the outputs obtained after applying the input *U*(*t*) on the real system and also in parallel on the fault-free and faulty NN models (Figure 5). Detection and diagnosis result from the generation of residuals *R*_*i*_(*t*),  *i* = 0,…, *p*, according to a decision block.

### 4.2. Fault Detection

During monitoring, the direct comparison of the system outputs *Y*(*t*) and the outputs *Y*_0_′(*t*) of fault-free model leads to residuals *R*_0_(*t*) = (*r*_*k*0_(*t*))  *k* = 1,…, *n* with

(4) $$\begin{array}{c} r_{k0}\left(t\right)=y_{k}\left(t\right)-y_{k}^{\prime }\left(t\right),\;k=1,\ldots ,n. \end{array}$$

**Figure 6:**
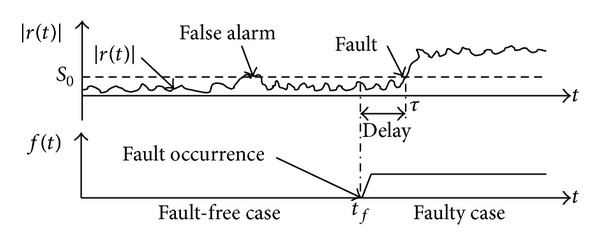
Fault detection using residual analysis.

The residual *R*_0_(*t*) provides information about faults for further processing. Fault detection is based on the evaluation of residuals magnitude. It is assumed that each residual *r*_*k*0_(*t*), *k* = 1,…, *n*, should normally be close to zero in the fault-free case, and it should be far from zero in the case of a fault. Thus, faults are detected by setting threshold *S*_*k*0_ on the residual signals (Figure 6, a single residual and a single fault are considered for simplicity). The analysis of residuals *r*_*k*0_(*t*) also provides an estimate *τ*_*k*_ of the time of occurrence *t*_*f*_ used for diagnosis issue. When several residuals are used, the estimate *τ* of the time of occurrence of faults is given by

(5) $$\begin{array}{c} \tau =\min\left\{\tau_{k},k=1,\ldots ,n\right\}. \end{array}$$

The faults are detected when the magnitude of one residual |*r*_*k*0_(*t*)| becomes larger than the threshold *S*_*k*0_:

(6) $$\begin{array}{c} \left|r_{k0}\left(t\right)\right|\leq S_{k0}:\text{no}\;\text{fault}\;\text{is}\;\text{detected}\;\text{at}\;\text{time}\;t,\\ \left|r_{k0}\left(t\right)\right|>S_{k0}:\text{a}\;\text{fault}\;\text{is}\;\text{detected}\;\text{at}\;\text{time}\;\;t. \end{array}$$

The main difficulty with this evaluation is that the measurement of the system outputs *y*_*k*_(*t*) is usually corrupted by disturbances (e.g., measurement noise). In practice, due to the modeling uncertainties and disturbances, it is necessary to assign large thresholds *S*_*k*0_ in order to avoid false alarms. Such thresholds usually imply a reduction of the fault detection sensitivity and can lead to no detections. In order to avoid such problems, one can run also the models of faulty behaviors from *t* = 0 and use the method described below. The idea is to evaluate the probability of the fault candidates at each time. A fault is detected when the probability of one model of faulty behaviors NNFM(*j*), *j* = 1,…, *p*, becomes larger than the probability of the fault-free model NNFM(0).

### 4.3. Proposed Method for Fault Diagnosis

The diagnosis results either from the usual thresholding technique or from the online determination of fault probabilities and confidence factors [39]. In the second method, the faulty models run simultaneously from time *t* = *τ* where *τ* is the fault detection time. Each model will behave according to a single fault candidate and the resulting behaviors will be compared with the collected data to provide a rapid diagnosis. In case of numerous fault candidates *f*_*i*_, *i* = 1,…, *p*, the output *Y*_*i*_′(*t*) = (*y*_*k*_′(*t*, *f*_*i*_, *τ*)) of the model NNFM(*i*) is compared with the measurement vector *Y*(*t*) to compute additive residual *R*_*i*_(*t*) = (*r*_*ki*_(*t*, *τ*)), *k* = 1,…, *n*. The most probable fault candidate is determined according to the comparison of all residuals *r*_*ki*_(*t*, *τ*), *k* = 1,…, *n*,  *i* = 1,…, *p*, resulting from the *n* outputs and *p* models of faults:

(7) $$\begin{array}{c} r_{ki}\left(t,\tau \right)=y_{k}\left(t\right)-y_{ki}^{\prime }\left(t,\tau \right). \end{array}$$

The introduction of probabilities to evaluate the significance of each residual and the reliability of the decision is another component of our approach. The proposed method uses a time window that can be sized according to the time requirement. Diagnosis with a large time window includes a diagnosis delay but will lead to a decision with a high confidence index. On the contrary single diagnosis with a small time window leads to early diagnosis but with a lower confidence index. To evaluate the probability of each fault candidate let us define *ρ*_*ki*_(*t*, *T*, *τ*) as the cumulative residuals over the sliding time interval [max⁡(0, *t* − *T*), *t*] of maximal size *T* (*T* stands for the size of time window):

(8) $$\begin{array}{c} \rho_{ki}\left(t,T,\tau \right)=\sqrt{\overset{t}{\underset{\max(0,t-T)}{\int }}{\left(r_{ki}\left(u,\tau \right)\right)}^{2}\cdot du}. \end{array}$$

Then, *D*_*i*_(*t*, *T*, *τ*) is the Euclidean norm of the vector Ω_*i*_(*t*, *T*, *τ*) = (*ρ*_*ki*_(*t*, *T*, *τ*)) of dimension *n*:

(9) $$\begin{array}{c} D_{i}\left(t,T,\tau \right)=\sqrt{\overset{k=n}{\underset{k=1}{\sum }}{\left(\rho_{ki}\left(t,T,\tau \right)\right)}^{2}}. \end{array}$$

*D*_*i*_(*t*, *T*, *τ*) is used to determine which is the most probable fault according to delayed or early diagnosis. Two particular cases are considered for *T* = *t* and *T* = 0.

The most probable fault at time *t* is given according to the a posteriori analysis of *D*_*i*_(*t*, *t*, *τ*) computed for the time interval [0, *t*]  (*T* = *t*):

(10) $$\begin{array}{c} i^{*}\left(0,t\right)=\underset{i}{\text{argmin}}\left\{D_{i}\left(t,t,\tau \right),i=1,\ldots ,p\right\}. \end{array}$$

The probability *P*_*i*_(*t*, *t*, *τ*) that the current fault is *f*_*i*_ will be given by

(11) Pi(t,t,τ)=1Di(t,t,τ)∑k=1k=p(1/Dk(t,t,τ))=[∑k=1,k≠ik=p(Di(t,t,τ)Dk(t,t,τ))+1]−1.

Immediate diagnosis results from the analysis of *D*_*i*_(*t*, 0, *τ*) computed at time *t* according to (*T* = 0):

(12) $$\begin{array}{c} i^{*}\left(t,t\right)=\underset{i}{\text{argmin}}\left\{D_{i}\left(t,0,\tau \right),i=1,\ldots ,p\right\}. \end{array}$$

In practical cases and in order to attenuate the effects of noise and outlaw values, the most probable fault candidate is determined according to the comparison of the cumulative residuals over a sliding time interval [max⁡(0, *t* − *T*), *t*] of maximal size *T*:

(13) $$\begin{array}{c} i^{*}\left(t-T,t\right)=\underset{i}{\text{argmin}}\left\{D_{i}\left(t,T,\tau \right),i=1,\ldots ,p\right\}. \end{array}$$

The probability *P*_*i*_(*t*, *T*, *τ*) that the current fault is *f*_*i*_ will be given by

(14) $$\begin{array}{c} P_{i}\left(t,T,\tau \right)={\left[\overset{k=p}{\underset{k=1,k\neq i}{\sum }}\left(\frac{D_{i}\left(t,T,\tau \right)}{D_{k}\left(t,T,\tau \right)}\right)+1\right]}^{-1}. \end{array}$$

The window size *T* is selected in order to satisfy real time requirements for rapid diagnosis. Let us mention that a confidence factor for diagnosis can also be worked out according to the probabilities *P*_*i*_(*t*, *T*, *τ*):

(15) CF(t,T,τ)=(max⁡⁡(Pi(t,T,τ)i=1,…,p)  −max⁡⁡(Pk(t,T,τ):k=1,…,p,k≠i)) ×(max⁡⁡(Pi(t,T,τ):i=1,…,p))−1.

The preceding method can also be combined with a thresholding technique to avoid the multiplication of residuals and to provide a reliable decision according to a hierarchical scheme. In a first stage, a small number of residuals are evaluated and analyzed. This stage leads to the determination of a subgroup of possible faults that have the same signature. Then, the fault probabilities are used within this subgroup in order to select the most probable fault candidate.

## 5. Application to Electropneumatic Actuator

### 5.1. Fault Detection

The residual vector *R*_0_(*t*) = (*r*_*k*0_(*t*)), *k* = 1, 2, is first considered for fault detection:

(16) $$\begin{array}{c} r_{10}\left(t\right)=X\left(t\right)-X^{\prime }\left(t\right),\\ r_{20}\left(t\right)=F\left(t\right)-F^{\prime }\left(t\right), \end{array}$$

where *X*′ and *F*′ are the outputs of the NN model of fault-free behaviors. The detection is obtained according to the comparison of residuals with appropriate thresholds. Three-valued signals are obtained (positive, negative, and zero). The thresholds were calculated according to the standard deviation of the residual for fault-free case [39]. Let us notice that the choice of constant or adaptive thresholds strongly influences the performance of the FDI system. The thresholds must be thoroughly selected. For the continuation of our work, the thresholds *S*_10_ = 100 · *σ*_1_ and *S*_20_ = 10 · *σ*_2_ are selected where *σ*_1_ and *σ*_2_ are the standard deviations obtained from the learning process.  Table 4 sums up the detection performances for the 19 types of faults according to the sign of the residual vector *R*_0_.

The evaluation of residual vector *R*_0_ leads to a first stage in detection and isolation: from Table 4, the faults *f*_2_, *f*_4_, *f*_11_, *f*_13_, and *f*_16_ have specific symptoms and can be directly isolated. Three groups of faults with similar symptoms can also be separated:

- group number 1 = {*f*_3_, *f*_6_, *f*_9_, *f*_12_, *f*_18_, *f*_19_};
- group number 2 = {*f*_1_, *f*_7_, *f*_10_, *f*_15_, *f*_17_};
- group number 3 = {Fault-free, *f*_5_, *f*_8_, *f*_14_}.

The faults in group 1 and group 2 are detected but not isolated because the signatures over *r*_10_ and *r*_20_ are similar. One can also notice that the faults in group 3 have the same signature as the fault-free behaviors. Thus faults in group 3 cannot be directly detected with residuals *r*_10_ and *r*_20_.

To illustrate our contribution, 3 simulations with faults are considered. The fault *f*_3_, that is, an incipient fault of group 1, is simulated during the time interval $\left[\begin{array}{cc} 487\;\text{s} & 1000\;\text{s} \end{array}\right]$; then *f*_15_, that is, an abrupt fault of group 2, is simulated during the time interval $\left[\begin{array}{cc} 451\;\text{s} & 1000\;\text{s} \end{array}\right]$; finally *f*_5_, that is, an incipient fault, is simulated during time interval [302 s, 1000 s]. All these simulations were realized by DABLIB models under Matlab Simulink. The detection thresholds are selected such that *S*_10_ = 10∗*σ*_1_ and *S*_20_ = 10∗*σ*_2_ with *σ*_1_ = 7.047∗10^−6^ and *σ*_2_ = 1.065∗10^−5^. According to the detection stage, the fault *f*_3_ is detected at time *τ* = 501 s and with a delay of 14 s the group 1 is also isolated. The fault *f*_15_ is detected at time *τ* = 458 s with a delay of 7 s and the group 2 is isolated. The fault *f*_5_ cannot be detected with the thresholding technique because it has the same signature as the fault-free behaviors.

### 5.2. Fault Diagnosis

Within each group, faults are not isolable with both residuals (*r*_10_ and *r*_20_). For this reason, the new technique proposed and described in Section 4.3 is used. For this purpose, models of faults corresponding to each fault candidates of the 3 groups are designed according to the historical data provided by DAMADICS benchmark. Each model NNFM(*i*) computes two estimated outputs *X*_*i*_′(*t*) and *F*_*i*_′(*t*) and the difference with measured data of the system leads to the residuals *r*_1*i*_(*t*) and *r*_2*i*_(*t*), *i* ∈ {3, 6, 9, 12, 18, 19, 1,7, 10, 15, 17, 5, 8, 14}:

(17) $$\begin{array}{c} r_{1i}\left(t\right)=X\left(t\right)-X_{i}^{\prime }\left(t\right),\\ r_{2i}\left(t\right)=F\left(t\right)-F_{i}^{\prime }\left(t\right). \end{array}$$

**Figure 7:**
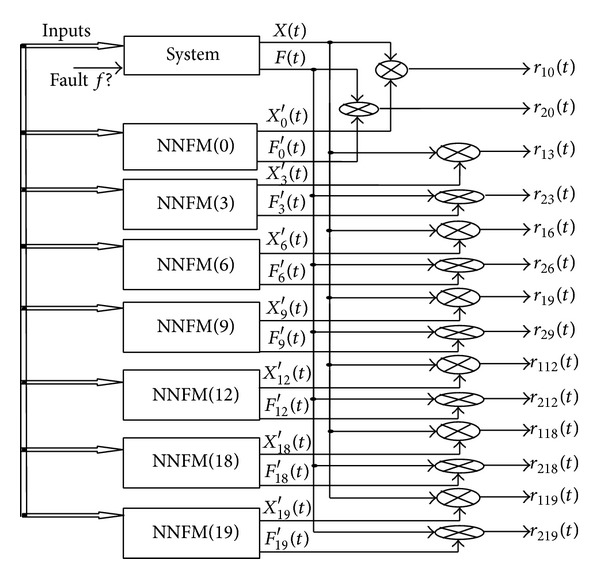
Fault diagnosis for a fault detected in group number 1.

**Figure 8:**
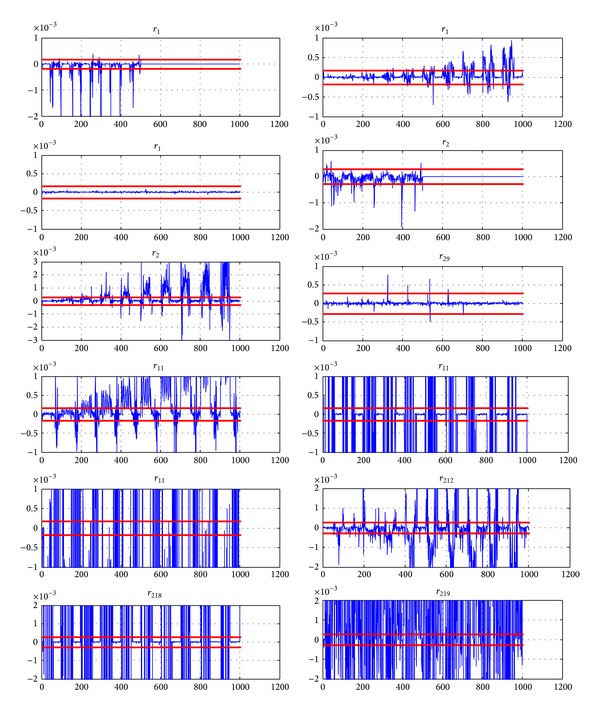
Residuals for group number 1 (*X*-label: time (s), *Y*-label: residual magnitude) when *f*_3_ is simulated from time *t* = 487 s.

When the fault *f*_3_ is simulated during the time interval [487 s  1000 s], all faulty models in group 1 are evaluated (Figure 7) and residuals from Figure 8 are obtained.

The residuals of Figure 8 are obtained. From time *τ* = 501 s, one can notice that only residuals *r*_13_ and *r*_23_ remain within the interval limited by thresholds *S*_10_ and *S*_20_.

The application of the usual thresholding method leads to partial isolation. The residuals *R*_6_, *R*_12_, *R*_18_, and *R*_19_ clearly exceed the thresholds for *t* > *τ*  (*τ* = 501 s) and thus the fault candidates *f*_6_, *f*_12_, *f*_18_, and *f*_19_ are eliminated. The residual *r*_29_ also exceeds the threshold in some points but these points can be interpreted as outlaws and the faults *f*_3_ and *f*_9_ are difficult to separate.

The second method leads to better results. Let us define the cumulative residuals *ρ*_1*i*_(*t*, *T*, *τ*), *ρ*_2*i*_(*t*, *T*, *τ*), and the distance *D*_*i*_(*t*, *T*, *τ*), according to (8) and (9). The application of the method described in Section 4.3 leads to the results in Table 5. Delayed diagnosis with a large time window is obtained according to (10).

The diagnosis results are reported in Table 5 for *T* = 1000 s. The column 5 of Table 5 shows that the probability for fault *f*_3_ is about 52% and the confidence factor for the diagnosis is about 51% according to (15). To conclude *f*_3_ is the most probable fault when residuals are analyzed within time interval [0, 1000 s].

**Figure 9:**
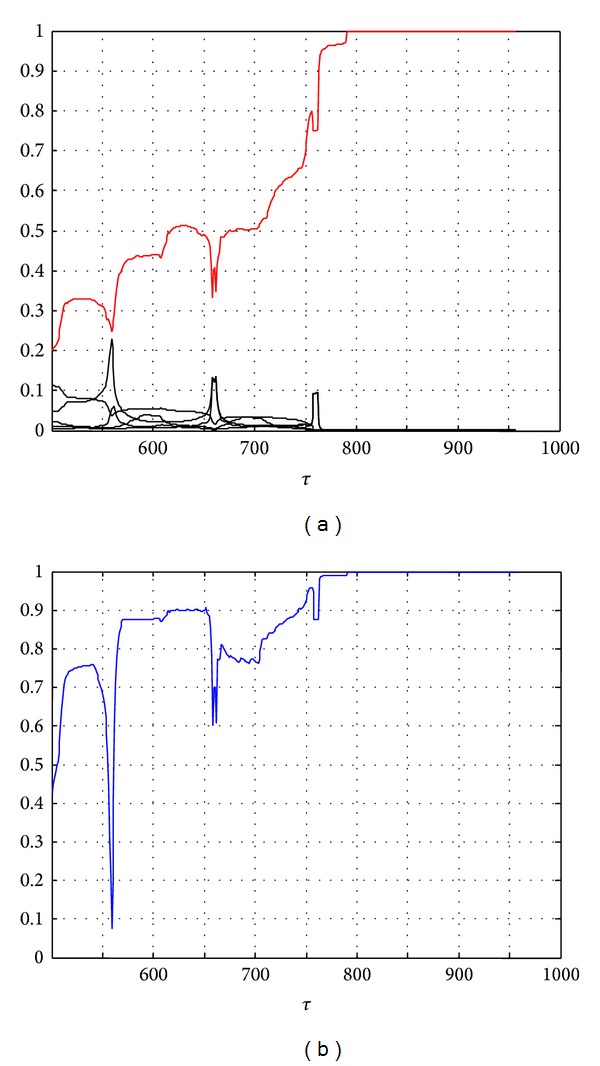
Performance evaluation for FDI of fault *f*_3_ (*X*-label: time (s), *Y*-label probability and confidence factor). (a) Probability *P*_3_(*t*, 50,501); (b) confidence factor CF(*t*, 50, 501).

Early diagnosis for fault *f*_3_ is also illustrated by selecting a small time interval with *T* = 50 s. For any *t* ∈ [0, 1000], the model with minimal distance to the origin (i.e., minimal value of *D*_*i*_(*t*, 50, *τ*)) corresponds to the most probable fault.  Figure 9 reports the probabilities of the fault candidates from the instant *τ* of detection versus time and also the confidence factor of the FDI decision. One can notice that the signals *P*_*j*_ and CF exhibit a specific frequency of 0.01 Hz that corresponds to the frequency of input.

In Figure 9(a), the curve above in red corresponds to the probability of the fault *f*_3_. This probability increases with time and reaches the value 1 at time *t* = *τ* + 290 = 791 s. It varies quickly during the decision phase $\left[\begin{array}{cc} 500\;\text{s} & 550\;\text{s} \end{array}\right]$. This illustrates the robustness of our method.  Figure 9(b) shows the variations of the confidence factor calculated by (15) and confirms that the *f*_3_ fault is the most probable fault.

**Figure 10:**
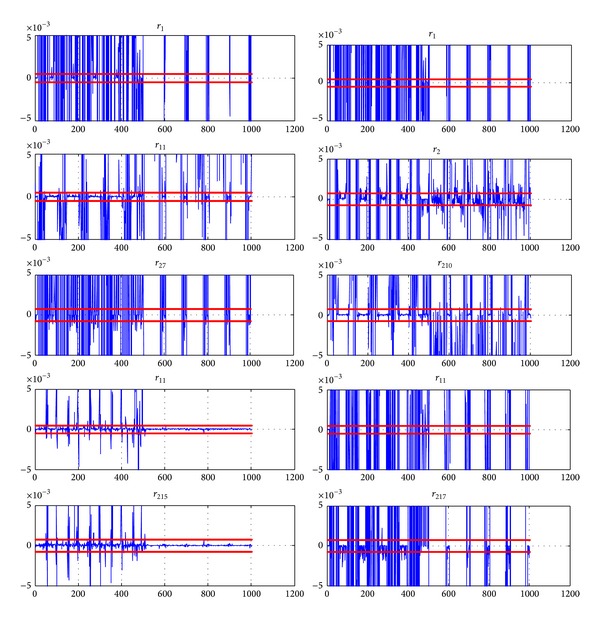
Residuals for group 2 (*X*-label: time (s), *Y*-label: residual magnitude) when *f*_15_ is simulated from time *t* = 451 s.

The fault *f*_15_ is also simulated during time interval $\left[\begin{array}{cc} 451\;\text{s} & 1000\;\text{s} \end{array}\right]$. This fault is detected at time 458 s and group 2 is isolated. Then all faulty models in group 2 are evaluated and residuals in Figure 10 are obtained.

The residuals of Figure 10 are obtained. From time *τ* = 458 s, one can notice that only residuals *r*_115_ and *r*_215_ remain within the interval limited by thresholds *S*_10_ and *S*_20_. So fault *f*_15_ is isolated.

The FDI method proposed is also applied to isolate *f*_15_. The application of the method described in Section 4.3 leads to the results in Table 6.

Table 6 reports the location of each model NNFM(*i*) in plan (*ρ*_1_, *ρ*_2_) and the distance *D*_*i*_(*t*, *t*, 458) at time *t* = 1000 s. The column 5 of Table 6 also reports the probabilities of each fault candidate according to (14). From this column one can conclude that the most probable fault is *f*_15_: the fault probability for *f*_15_ is about 96%. In the same time the probabilities of the other faults do not exceed 3%. Such indicators provide a confidence factor for the diagnosis about 96% according to (15).

**Figure 11:**
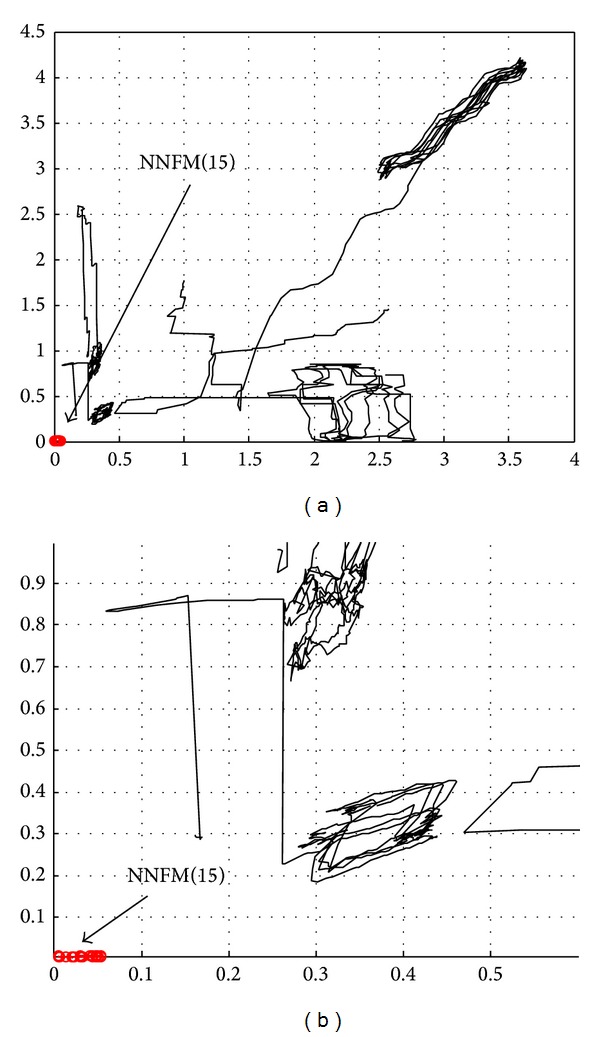
Early diagnosis for fault *f*_15_ (*X*-label: *ρ*_1_, *Y*-label: *ρ*_2_): (a) location of the models NNFM(*i*); (b) details.

Early diagnosis of fault *f*_15_ is illustrated by selecting a small time interval with *T* = 50 s. For any *t* ∈ [0, 1000], the model with minimal distance to the origin corresponds to the most probable fault. In Figure 11(a), all trajectories are reported; the trajectory for model NNFM(15) is highlighted.  Figure 11(b) plots details about the trajectory for model NNFM(15).

**Figure 12:**
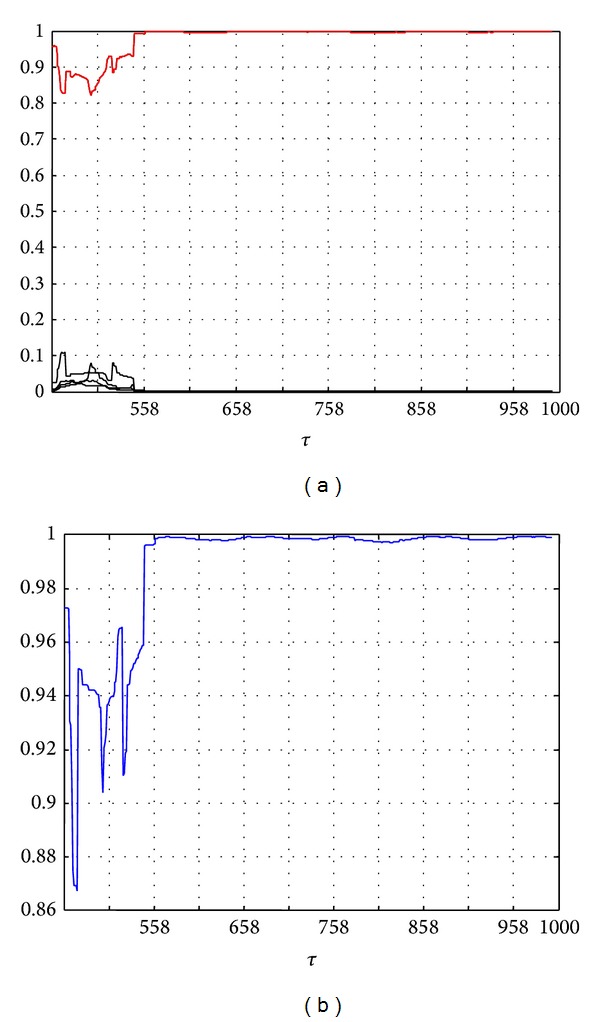
Performance evaluation for FDI of fault *f*_15_ (*X*-label: time (s), *Y*-label probability and confidence factor). (a) Probability *P*_15_(*t*, 50,458); (b) confidence factor CF(*t*, 50,458).

The trajectory corresponding to NNFM(15) remains near origin in comparison to the other trajectories. One can conclude that the fault candidate *f*_15_ is the most probable fault. The repartition of the cumulative residuals in plan (*ρ*_1_, *ρ*_2_) confirms the significance of both outputs *X*(*t*) and *F*(*t*) to design residuals (we can notice that cumulative residuals *ρ*_1*i*_(*t*, *T*, *τ*) and *ρ*_2*i*_(*t*, *T*, *τ*) cover the positive part of plan (*ρ*_1_, *ρ*_2_)).  Figure 12 reports the probabilities of the fault candidates from the instant *τ* of detection versus time and also the confidence factor of the FDI decision.

In Figure 12(a), the curve above corresponds to the probability of the fault *f*_15_. This probability increases very quickly and reaches the value 1 at time *t* = *τ* + 100 = 558 s.  Figure 12(b) shows the variations of the confidence factor calculated by (15) and confirms that the *f*_15_ fault is the most probable fault. One can notice that the confidence factor for the isolation of fault *f*_15_ reaches quickly the value 1 in comparison with fault *f*_3_: the reason is that *f*_15_ is an abrupt fault whereas *f*_3_ is an incipient one.

**Figure 13:**
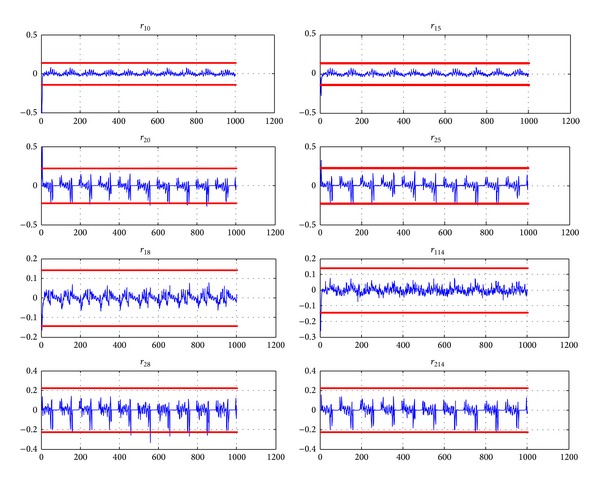
Residuals for group 3 (*X*-label: time (s), *Y*-label: residual magnitude) when *f*_5_ is simulated from time *t* = 302 s to 1000 s.

The fault *f*_5_ is also simulated during time interval $\left[\begin{array}{cc} 302\;\text{s} & 1000\;\text{s} \end{array}\right]$. This fault cannot be detected with the thresholding technique: the residuals in Figure 13 are obtained and one can notice that no residual from group 3 overcomes the thresholds previously defined.

**Figure 14:**
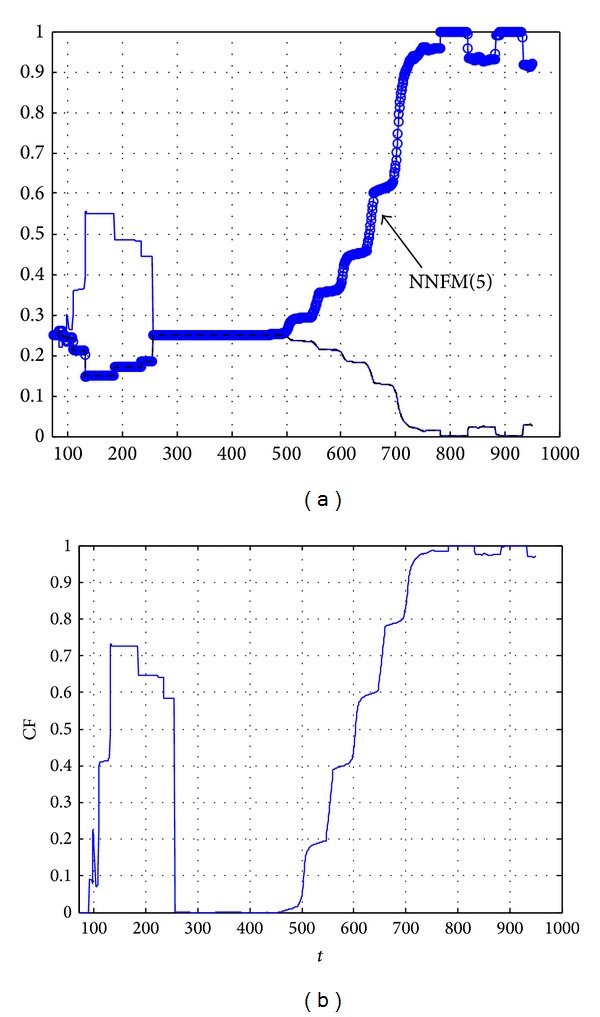
Performance evaluation for FDI of fault *f*_5_ (*X*-label: time (s), *Y*-label probability and confidence factor). (a) Probability *P*_5_; (b) confidence factor CF.

In this case, detection and isolation are obtained in a single stage by considering simultaneously all residuals for models in group number 3 (i.e., *R*_0_, *R*_5_, *R*_8_, and *R*_14_). The probabilities of the models NNFM(0), NNFM(5), NNFM(8), and NNFM(14) are reported in Figure 14(a). In this figure, one can notice that the probability of model NNFM(0) is clearly the largest one from *t* = 100 s to *t* = 300 s (bleu line), then from *t* = 300 s to *t* = 500 s; the probabilities of all models are very similar and finally the probability of model NNFM(5) increases from time *t* = 500 s (curve with blue circles). The confidence factor reported in Figure 14(b) illustrates that the decisions provided by the FDI system are reliable in intervals $\left[\begin{array}{cc} 100\;\text{s} & 300\;\text{s} \end{array}\right]$ and $\left[\begin{array}{cc} 500\;\text{s} & 1000\;\text{s} \end{array}\right]$.

### 5.3. Discussion

Table 7 reports some conclusion concerning the detection and diagnosis of faults for the DAMADICS benchmark and according to the considered method. Results are detailed (1) for the fault detection with thresholds (according to the evaluation of residual *R*_0_); (2) for the fault isolation with thresholds (according to the evaluation of residuals *R*_0_ to *R*_19_); (3) for the fault detection and isolation with probability and confidence factor computation (according to the evaluation of residuals *R*_0_ to *R*_19_). 84% of the fault candidates are detected with the thresholding method. The delay to detection never exceeds 30 s. But faults in group 3 are not detectable with the considered thresholds. Decreasing the detection thresholds improves detection results but leads also to false alarms and fault *f*_14_ remains undetectable. Some faults are detected but cannot be isolated with thresholds (e.g., *f*_3_ and *f*_9_): isolation succeeds for 63% of the fault candidates with the thresholding method. In comparison, the computation of fault probabilities and confidence factor leads to the detection and isolation of all faults (for the considered example). In a few cases, the confidence factor is near 0.5 and the decision is not considered as reliable. The computation effort with the proposed method is to run several (up to 6) models in parallel.

## 6. Conclusion

The proposed FDI scheme combined the design of neural networks to model fault-free and faulty behaviors of industrial systems (residuals generation by using thresholding method for isolation) with a probabilistic method (evaluating the fault probability and the confidence on decision). The results are compared with a usual thresholding method. Both techniques give correct decisions in many cases. However, the results obtained with the method based on the computation of the probabilities are better and the reliability of the decision is also explicitly evaluated. In particular the proposed method does not require computing thresholds for detection and isolation and as a consequence is easier to use for incipient faults.

The systematic design of fault-free and faulty models based on NNs has been proved to be suitable for early detection and diagnosis issues in case of nonlinear systems. The application of the proposed method on the DAMADICS benchmark illustrates also the performance of the proposed FDI approach.

From our point of view, the main limitation of the proposed method is the rapid increase of the computational effort when numerous fault candidates and numerous outputs are considered. To reduce this effort, one can notice that some residuals contain useful information for FDI and others are quite useless. Based on the evaluation of a confidence factor for each residual, we will study a method to select the more reliable residuals. Another drawback is that the proposed method requires the design of models that include the influence of faults. The strength and size of faults also can influence the model behavior. For these reasons, the method must be carefully applied depending on the system under conditions. Our future works are also to validate this technique by applying it on other systems with various operating conditions and various faults.

## Figures and Tables

**Table 1:** Set of faults specified for Benchmark actuator.

Fault	Description	Time development			
		**S**	**M**	**B**	Incipient
*f*_1_	Valve clogging	∗	∗	∗	
*f*_2_	Valve or valve seat sedimentation			∗	∗
*f*_3_	Valve or valve seat erosion				∗
*f*_4_	Increasing of valve or bushing friction				∗
*f*_5_	External leakage				∗
*f*_6_	Internal leakage (valve tightness)				∗
*f*_7_	Medium cavity or critical flow	∗	∗	∗	
*f*_8_	Twisted servomotor's rod	∗	∗	∗	
*f*_9_	Servomotor's housing or terminals tightness				∗
*f*_10_	Servomotor's diaphragm perforation	∗	∗	∗	
*f*_11_	Servomotor's spring fault			∗	∗
*f*_12_	Electropneumatic transducer fault	∗	∗	∗	
*f*_13_	Rod displacement sensor fault	∗	∗	∗	∗
*f*_14_	Pressure sensor fault	∗	∗	∗	
*f*_15_	Positioner spring fault			∗	
*f*_16_	Positioner lever fault	∗	∗	∗	
*f*_17_	Positioner supply pressure drop			∗	∗
*f*_18_	Unexpected change of pressure difference	∗	∗	∗	∗
*f*_19_	Fully or partly opened bypass valves	∗	∗	∗	

**Table 2:** Input and output variables for actuator.

Input	Range	Unit	Description
CV	[0,1]	—	Control signal from external PI controller
*P*_1_	[2000,4*e* + 6]	Pa	Inlet liquid pressure
*P*_2_	[2000,4*e* + 6]	Pa	Outlet liquid pressure
*T*_1_	[30,110]	C°	Liquid temperature

Output	Range	Unit	Description

*X*	[0,1]	—	Position of the rod
*F*	[0,1]	—	Average flow

**Table 3:** Structure selection for NNFM(0).

NNFM	Hidden layer 1	Hidden layer 2	Output layer	MSE
(6, 3, 2)	6	3	2	3.3 ∗ 10^−4^
(10, 8, 2)	10	8	2	1.49 ∗ 10^−4^
(21, 12, 2)	21	12	2	3.91 ∗ 10^−4^
(26, 26, 2)	26	26	2	4.84 ∗ 10^−6^

**Table 4:** Fault detection with residuals *r*_10_ and *r*_20_.

Faults	Residuals				
	*r*_10_ > 0	*r*_10_ < 0	*r*_20_ > 0	*r*_20_ < 0	Group number
Fault-free	0	0	0	0	3
*f*_1_	1	1	1	1	2
*f*_2_	0	0	1	0	Isolated
*f*_3_	0	0	0	1	1
*f*_4_	0	0	1	1	Isolated
*f*_5_	0	0	0	0	3
*f*_6_	0	0	0	1	1
*f*_7_	1	1	1	1	2
*f*_8_	0	0	0	0	3
*f*_9_	0	0	0	1	1
*f*_10_	1	1	1	1	2
*f*_11_	1	1	1	0	Isolated
*f*_12_	0	0	0	1	1
*f*_13_	0	1	0	1	Isolated
*f*_14_	0	0	0	0	3
*f*_15_	1	1	1	1	2
*f*_16_	1	0	0	1	Isolated
*f*_17_	1	1	1	1	2
*f*_18_	0	0	0	1	1
*f*_19_	0	0	0	1	1

**Table 5:** Delayed diagnosis for fault *f*_3_ with *T* = *t* = 1000 s.

Model of faults NNFM(*i*)				
Fault candidate	*ρ*_1*i*_(1000,1000,501)	*ρ*_1*i*_(1000,1000,501)	*D*_*i*_(1000,1000,501)	*P*_*i*_(1000,1000,501)
*f*_3_	*0.01 *	*0.90 *	*0.90 *	*0.51 *
*f*_6_	0.03	6.48	6.48	0.07
*f*_9_	0.09	1.84	1.84	0.25
*f*_12_	2.71	5.73	6.34	0.07
*f*_18_	0.03	9.92	9.92	0.04
*f*_19_	0.03	13.13	13.13	0.03

**Table 6:** Delayed diagnosis for fault *f*_15_ with *T* = *t* = 1000 s.

Model of faults NNFM(*i*)				
Fault Candidate	*ρ*_1*i*_(1000,1000,458)	*ρ*_1*i*_(1000,1000,458)	*D*_*i*_(1000,1000,458)	*P*_*i*_(1000,1000,458)
*f*_1_	8.93	4.09	9.82	0.007
*f*_7_	10.38	12.52	16.27	0.004
*f*_10_	1.23	1.44	1.90	0.037
*f*_15_	*0.06 *	*0.04 *	*0.07 *	*0.935 *
*f*_17_	1.69	4.50	4.81	0.015

**Table 7:** Comparison of FDI methods (+: decision is correct; −: decision is wrong).

Faults	Technique		
	Detection with threshold	Isolation with threshold	FDI with confidence factor
*f*_1_	+	−	+
*f*_2_	+	+	+
*f*_3_	+	−	+
*f*_4_	+	+	+
*f*_5_	−	−	+
*f*_6_	+	−	+
*f*_7_	+	+	+
*f*_8_	−	−	+
*f*_9_	+	−	+
*f*_10_	+	+	+
*f*_11_	+	+	+
*f*_12_	+	+	+
*f*_13_	+	+	+
*f*_14_	−	−	+
*f*_15_	+	+	+
*f*_16_	+	+	+
*f*_17_	+	+	+
*f*_18_	+	+	+
*f*_19_	+	+	+
